# Complete Genome Sequences of the Probiotic Lactic Acid Bacteria *Lactobacillus helveticus* D75 and D76

**DOI:** 10.1128/genomeA.01552-17

**Published:** 2018-03-15

**Authors:** V. A. Toropov, T. Y. Vakhitov, O. N. Shalaeva, E. K. Roshchina, S. I. Sitkin

**Affiliations:** a State Research Institute of Highly Pure Biopreparations of the FMBA of the Russian Federation, St. Petersburg, Russia

## Abstract

*Lactobacillus helveticus* D75 and D76 were isolated from the intestinal tract of a healthy child. Both strains possess symbiotic, probiotic, and antagonistic activities. We have sequenced and annotated the whole genomes of *L. helveticus* D75 and D76 and have conducted a preliminary genome comparative analysis.

## GENOME ANNOUNCEMENT

*Lactobacillus helveticus* D75 and D76 have pronounced fermentative and probiotic activities (1). It was important to have information that was as complete as possible about the genomes of *L. helveticus* strains D75 and D76 to understand the mechanisms of regulation of their probiotic activity and syntrophic interactions.

*L. helveticus* D75 and D76 were grown on de Man-Rogosa-Sharpe (MRS) medium (2) at 37°C up to the mid-exponential-growth phase. The modified method of cell lysis of Gram-positive bacteria and the conventional method of DNA extraction with organic solvents were used to obtain the chromosomal DNA (3). The cell lysis consisted of two phases. Initially, a Tris-EDTA buffer containing mutanolysin (final concentration, 300 U/ml) and lysozyme (final concentration, 2 mg/ml) was added to the cell pellet, and the resulting mixture was incubated at 37°C for 1 h. Then, a solution containing sodium dodecyl sulfate (final concentration, 1.5%) and proteinase K (final concentration, 1 mg/ml) was added, and the mixture was incubated at 50°C for 1 h.

Both genomic DNAs were sequenced using the PacBio RS II platform (Macrogen, Inc., Republic of Korea) (4–7). Genome libraries consisting of 150,292 and 166,471 reads with *N*_50_ values of 22,778 bp and 10,700 bp were obtained for *L. helveticus* D75 and D76, respectively. The HGAP algorithm in the SMRT Analysis pipeline version 2.3.0 was used to assemble the genomes of *L. helveticus* D75 and D76 from PacBio RS raw reads (8). The lengths of the whole genomes obtained were 2,053,066 bp (with 422× read multiplicity) and 2,058,319 bp (with 375× read multiplicity) for the *L. helveticus* D75 and D76 strains, respectively.

The genome annotations of *L. helveticus* D75 and D76 were done using the Prokaryotic Genome Annotation Pipeline (PGAP) algorithm of the National Center for Biotechnological Information (NCBI) (9). The annotated genome of *L. helveticus* D75 contained 2,092 coding sequences (CDS), with 1,693 protein-coding genes, 64 tRNA genes, and 15 rRNA genes. The total number of pseudogenes was 317. *L. helveticus* D76 includes 2,068 CDS, with 1,986 protein-coding genes, 64 tRNA genes, and 15 rRNA genes. The total number of pseudogenes was 265. The BLASTn algorithm was applied for preliminary genome comparative analysis of *L. helveticus* D75 and D76 with the genomes of *L. helveticus* DPC4571 and *L. helveticus* R0052, which were annotated and deposited in GenBank (10, 11). The *L. helveticus* D75 and D76 strains had 99.18% identity (90.65% coverage) and 97.73% identity (80.45% coverage) to the genomes of the DPC4571 and R0052 strains, respectively.

Calculating the average nucleotide identity (ANI) (12–14) showed that the genome sequences of *L. helveticus* D75 and D76 were 99.22% identical (with 76.3% coverage of the genome) to the genomes of *L. helveticus* species. Therefore, D75 and D76 strains, previously attributed to *L. acidophilus* species (based both on phenotype and 16S rRNA genetic analysis), were reclassified as *L. helveticus* D75 and D76.

### Accession number(s).

These whole-genome shotgun projects have been deposited in DDBJ/ENA/GenBank under the accession no. CP020029 (*L. helveticus* D75) and CP016827 (*L. helveticus* D76). The versions described in this paper are the first versions.

## References

[1] Vakhitov TY, Verbitskaya NB, Dobrolezh OV, Polevaya EV, Kobatov AI 2013 The effect of probiotic and pathogenic bacteria metabolites on antagonistic activity of *Lactobacillus acidophilus* D No. 75. Sci J KubSAU 92:339–357.

[2] De Man JC, Rogosa M, Sharpe ME 1960 A medium for the cultivation of lactobacilli. J Appl Bacteriol 23:130–135. doi:10.1111/j.1365-2672.1960.tb00188.x.

[3] Bollet C, Gevaudan MJ, de Lamballerie X, Zandotti C, de Micco P 1991 A simple method for the isolation of chromosomal DNA from Gram positive or acid-fast bacteria. Nucleic Acids Res 19:1995. doi:10.1093/nar/19.8.1955. PMC328140 2030980

[4] Khan IU, Yadav JS 2004 Development of a single-tube, cell lysis-based, genus-specific PCR method for rapid identification of mycobacteria: optimization of cell lysis, PCR primers and conditions, and restriction pattern analysis. J Clin Microbiol 42:453–457. doi:10.1128/JCM.42.1.453-457.2004. 14715804 PMC321669

[5] Van Soolingen D, Hermans PW, de Haas PE, Soll DR, van Embden JD 1991 Occurrence and stability of insertion sequences in *Mycobacterium tuberculosis* complex strains: evaluation of an insertion sequence-dependent DNA polymorphism as a tool in the epidemiology of tuberculosis. J Clin Microbiol 29:2578–2586. 1685494 10.1128/jcm.29.11.2578-2586.1991 PMC270376

[6] Rhoads A, Au KF 2015 PacBio sequencing and its applications. Genomics Proteomics Bioinformatics 13:278–289. doi:10.1016/j.gpb.2015.08.002. 26542840 PMC4678779

[7] Zhu L, Zhong J, Jia X, Liu G, Kang Y, Dong M, Zhang X, Li Q, Yue L, Li C, Fu J, Xiao J, Yan J, Zhang B, Lei M, Chen S, Lv L, Zhu B, Huang H, Chen F 2016 Precision methylome characterization of *Mycobacterium tuberculosis* complex (MTBC) using PacBio single-molecule real-time (SMRT) technology. Nucleic Acids Res 44:730–743. doi:10.1093/nar/gkv1498. 26704977 PMC4737169

[8] Chin CS, Alexander DH, Marks P, Klammer AA, Drake J, Heiner C, Clum A, Copeland A, Huddleston J, Eichler EE, Turner SW, Korlach J 2013 Nonhybrid, finished microbial genome assemblies from long-read SMRT sequencing data. Nat Methods 10:563–569. doi:10.1038/nmeth.2474. 23644548

[9] Tatusova T, DiCuccio M, Badretdin A, Chetvernin V, Nawrocki EP, Zaslavsky L, Lomsadze A, Pruitt KD, Borodovsky M, Ostell J 2016 NCBI prokaryotic genome annotation pipeline. Nucleic Acids Res 44:6614–6624. doi:10.1093/nar/gkw569. 27342282 PMC5001611

[10] Callanan M, Kaleta P, O’Callaghan J, O’Sullivan O, Jordan K, McAuliffe O, Sangrador-Vegas A, Slattery L, Fitzgerald GF, Beresford T, Ross RP 2008 Genome sequence of *Lactobacillus helveticus*, an organism distinguished by selective gene loss and insertion sequence element expansion. J Bacteriol 190:727–735. doi:10.1128/JB.01295-07. 17993529 PMC2223680

[11] Tompkins TA, Barreau G, Broadbent JR 2012 Complete genome sequence of *Lactobacillus helveticus* R0052, a commercial probiotic strain. J Bacteriol 194:6349. doi:10.1128/JB.01638-12. 23105080 PMC3486388

[12] Arahal DR 2014 Whole-genome analyses: average nucleotide identity. Methods Microbiol 41:103–122.

[13] Goris J, Konstantinidis KT, Klappenbach JA, Coenye T, Vandamme P, Tiedje JM 2007 DNA-DNA hybridization values and their relationship to whole-genome sequence similarities. Int J Syst Evol Microbiol 57:81–91. doi:10.1099/ijs.0.64483-0. 17220447

[14] Konstantinidis KT, Tiedje JM 2005 Genomic insights that advance the species definition for prokaryotes. Proc Natl Acad Sci U S A 102:2567–2572. doi:10.1073/pnas.0409727102. 15701695 PMC549018

